# The Richmond Agitation-Sedation Scale modified for palliative care inpatients (RASS-PAL): a pilot study exploring validity and feasibility in clinical practice

**DOI:** 10.1186/1472-684X-13-17

**Published:** 2014-03-31

**Authors:** Shirley H Bush, Pamela A Grassau, Michelle N Yarmo, Tinghua Zhang, Samantha J Zinkie, José L Pereira

**Affiliations:** 1 Division of Palliative Care, Department of Medicine, University of Ottawa, Ottawa, Canada; 2 Bruyère Research Institute, Ottawa, Canada; 3 Department of Palliative Care, Bruyère Continuing Care, 43 Bruyère Street, K1N 5C8 Ottawa, Ontario, Canada; 4 Ottawa Hospital Research Institute, Methods Centre, Ottawa, OT, Canada

**Keywords:** Palliative care, Richmond Agitation-Sedation Scale (RASS), Palliative sedation, Agitation, Delirium

## Abstract

**Background:**

The Richmond Agitation-Sedation Scale (RASS), which assesses level of sedation and agitation, is a simple observational instrument which was developed and validated for the intensive care setting. Although used and recommended in palliative care settings, further validation is required in this patient population. The aim of this study was to explore the validity and feasibility of a version of the RASS modified for palliative care populations (RASS-PAL).

**Methods:**

A prospective study, using a mixed methods approach, was conducted. Thirteen health care professionals (physicians and nurses) working in an acute palliative care unit assessed ten consecutive patients with an agitated delirium or receiving palliative sedation. Patients were assessed at five designated time points using the RASS-PAL. Health care professionals completed a short survey and data from semi-structured interviews was analyzed using thematic analysis.

**Results:**

The inter-rater intraclass correlation coefficient range of the RASS-PAL was 0.84 to 0.98 for the five time points. Professionals agreed that the tool was useful for assessing sedation and was easy to use. Its role in monitoring delirium however was deemed problematic. Professionals felt that it may assist interprofessional communication. The need for formal education on why and how to use the instrument was highlighted.

**Conclusion:**

This study provides preliminary validity evidence for the use of the RASS-PAL by physicians and nurses working in a palliative care unit, specifically for assessing sedation and agitation levels in the management of palliative sedation. Further validity evidence should be sought, particularly in the context of assessing delirium.

## Background

Best practices in palliative sedation (PS) include the use of standardized instruments to assess the level of sedation and enhance monitoring and documentation [1-4]. The European Association for Palliative Care’s (EAPC) Expert Working Group on Palliative Sedation recommends the Richmond Agitation-Sedation Scale (RASS) or similar tools to assess sedation and distress levels in palliative care patients with lowered consciousness [1].

The original RASS, developed for adult intensive care unit (ICU) patients, demonstrates strong inter-rater reliability in that setting [5-7]. It is a simple observational instrument assessing levels of sedation and agitation that requires no patient input [8] with scores ranging from +4 (representing an overtly combative patient) to -5, representing a patient who cannot be aroused by either voice or physical stimulation. A recent study found the RASS to be considerably less time consuming and easier to use than two other similar instruments [8]. A modified version of the RASS administered daily reported good sensitivity and specificity for incident delirium in veterans [9]. Although the RASS is currently used in many palliative care settings [8,10-12], there has only been one recently published report exploring its reliability in Spanish patients with advanced cancer [13]. Refractory agitated delirium at the end of life is the most frequent indication for palliative sedation [2,10,14]. The goal of this study was to investigate the validity and feasibility of the RASS-PAL, a version of the RASS slightly modified for palliative care populations, in patients experiencing agitated delirium or receiving PS.

## Methods

A prospective exploratory study, using a mixed methods approach, was undertaken. The study was approved by the institutions’ Research Ethics Boards (Bruyère Continuing Care Research Ethics Board and Ottawa Hospital Research Ethics Board). Informed consent from patients was waivered. Participating health care professionals (HCPs) provided informed written consent and received a short education session outlining the RASS-PAL administration procedure and scoring schedule. (The RASS had not been previously used on the PCU). Basic HCP demographic and patient information was collected.

The study was conducted on the 36-bed inpatient Palliative Care Unit (PCU) during working hours, Monday to Friday. Consecutive patients with an agitated delirium or receiving continuous PS were identified by attending physicians. On the PCU, if a patient screens positive on the Nursing Delirium Screening Scale (Nu-DESC) [15], the attending confirms the diagnosis using the Confusion Assessment Method (CAM) [16] and conducts a clinical evaluation. An a priori decision was made to conduct the study in ten patients and to analyze the data. This number of patients was felt to be adequate in terms of collecting sufficient data points for inter-rater exploration in this pilot study.

Patients were evaluated every hour for the first 4 hours (T1, T2, T3 and T4) on Day 1 and then 24 hours after T4 (T5). At each time point, one physician and one bedside nurse (BSN) and/or Practice Support Nurse (PSN) observed the patient simultaneously but independently, to remain blinded to the others’ results. The intent was for the same physician/nurse team to perform all five of the assessments for an individual patient. After completing their score raters could discuss their respective scores, but not alter it, and adjust patient management if needed. Intraclass correlation coefficients (ICC) and corresponding 95% confidence intervals were calculated to measure the degree of agreement among raters for T1-T5. SAS version 9.1 (SAS Institute Inc., Cary, NC, USA) was used for the statistical analysis.

Initially minor modifications to the RASS were made; the scale remained unchanged but the descriptors related to ‘pulling tubes’ and ‘fighting the ventilator’ were modified as these are usually not applicable in the palliative care context. Although the initial plan was to conduct a single study, a second study was conducted when the results showed that further minor modifications were required prior to validating the RASS-PAL in the palliative care context [17]. This modification to the research plan was approved by the Research Ethics Boards. This component of the study is referred to as the “Follow-up” study. In the “Initial” study, four palliative care physicians, eight BSN and one PSN participated. The intraclass correlation coefficient ranged from 0.76 to 0.95. However themes from interview data revealed the need to exclude ‘physical stimulation’ as HCPs felt this to be inappropriate. The need to standardize the scoring of patients with episodes of mixed delirium (with both agitated and hypoactive features) was also identified, as was the need for more education on the role of the tool.

In the “Follow-up” study, the instrument was modified so as to clarify that ‘any movement’ refers to eye or body, and ‘physical stimulation’ was changed to ‘gentle’ physical stimulation. For the ‘Procedure for Assessment’, the observation period was changed from 30 seconds to 20 seconds (as utilized by Ely) [6]. Clarification was also added on how to score a patient with a mixed type delirium. (See Additional file 1: Figure S1 and Additional file 2: Table S2 for the RASS-PAL tool). Otherwise, the research methods in the “Follow-Up” study were the same as in the “Initial” study.

Towards the final stages of the study, participating HCPs completed a brief written survey consisting of seven questions with a 5-point Likert Scale (1 = strongly disagree, 5 = strongly agree). Questions comprised the ease of using the RASS-PAL instrument, how well the scale measured sedation and agitation, whether the scale assisted in the monitoring of patients for sedation purposes and patients with an agitated delirium, and whether the scale improved patient care and aided health care professional communication. Trained research assistants (MY, SZ) conducted the HCP semi-structured interviews (10-15 minute duration). These were audio-recorded, transcribed verbatim and then cleared of any identifying information. Each interview was guided by four questions: the clinical advantages and limitations of the RASS-PAL in a palliative care inpatient population, its role in patient care and suggestions to improve it.

Transcribed interviews were coded manually and then imported into NVivo version 9 (QSR International, Doncaster, Victoria, Australia) for data analysis. As the interviews followed a very brief interview guide designed predominantly to gather evidence for face and construct validity for the RASS-PAL, we drew on a thematic analysis strategy to access accounts of how HCPs responded to the tool overall [18-20]. Two members of the research team (SB and MY) thematically coded an initial subset of three interview transcripts independently before reviewing their line by line coding together and building working definitions of each of their themes. Drawing on early themes grounded in the data, the team went back into the data to independently code the remaining data. All transcripts were reviewed to ensure coding consensus. Any differences in coding were reviewed and discussed until the team reached consensus. An external qualitative researcher (PG) then reviewed and applied the fully conceptualized coding tree to the data to ensure that codes had been consistently applied across the whole data set. An audit trail was utilized across the study, and detailed and comprehensive descriptions were used to describe and document the themes as they arose in the analysis [21]. A study journal was also kept, documenting any relevant findings or insights as the study progressed.

## Results

Results of the “Follow-up” study are reported here. Five palliative care physicians, eight nurses (seven BSNs and one PSN) participated. Three physicians had 15 years or more experience working in palliative care, one had 6-9 years, and one had 2-5 years. Three nurses had 15 years or more experience, two had 6-9 years, and three had 2-5 years. Of the patients identified for the study, one was excluded due to deafness and impaired vision as they were not able to respond to verbal commands or visual cues [5,6]. The included patients (eight male and two female) had a mean age of 73.6 years (range 56-88 years). Nine patients had metastatic cancer, and one patient had end-stage Amyotrophic Lateral Sclerosis (ALS).

**Figure 1:**
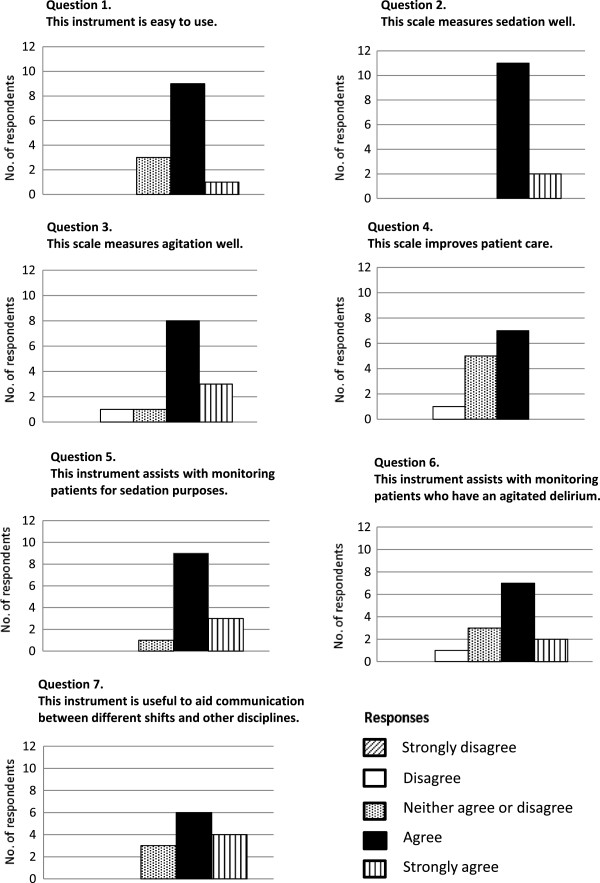
Health Care Professional (HCP) survey results.

Table 1 shows the RASS-PAL scores for the ten patients. The RASS-PAL scores of patient ID10 were excluded from the final analysis due to significant time lags between raters’ assessments, as inter-rater reliability has been shown to be reduced if the time between paired assessments is more than 15 minutes [8]. There were 35 RASS-PAL observation time points with two to three raters present for the nine included patients; with a total of 89 assessment events. The inter-rater intraclass correlation coefficients (ICC) for T1-T5 for these nine patients were 0.98, 0.84, 0.94, 0.97 and 0.98 respectively (Table 2). The results of the 13 completed surveys are shown in Figure 1 with data from the single PSN reported as aggregate data with the BSN data to ensure anonymity.

**Table 1:** RASS-PAL scores

**Patient ID**	**PPS**	**Indication**	**RASS-PAL Scores**				
			**(as rated by HCP1, HCP2, HCP3)**				
			**T1**	**T2**	**T3**	**T4**	**T5**
1	30	PS	n.d., −5, − 5	−3, −4, −5	n.d., −4, −5	−5, −5, −5	Patient died
3	30	Agitated delirium	−1, −1, −1	n.d., −1, −1	n.d., n.d., −1	−1, −1,-1	n.d., −1, −1
4	30	Agitated delirium	n.d., −5, −2	−5, −5, −4	n.d., −5, −4	n.d., n.d., n.d.	Patient died
5	20	Agitated delirium	1, 1, n.d.	n.d., n.d., n.d.	n.d., 0, 0	n.d., 0, 0	n.d., −3, n.d.
6	10	Agitated delirium	1, 0, 0	−1, 0, n.d.	−4, −5, −5	−1, −1, n.d.	n.d., −5, −5
7	10	PS	−2, n.d., −2	−2, n.d., −1	−5, n.d., −4	−5, n.d., −4	Patient died
8	10	PS	−3, −3, −3	−3, −5, −3	n.d., −4, −4	−2, n.d., −3	−5, n.d., −5
9	20	PS	−2, −2, −2	n.d., n.d., 0	n.d., n.d., 1	n.d., n.d., 1	0, 0, n.d.
10	20	Agitated delirium	2, 1, 1	1, −3, −3	n.d., −2, 1	1, −3, 1	1, −1, n.d.
11	40	Agitated delirium	−3, −3, −3	0, 0, 0	−1, 0, 0	−3, 0, −3	−1, 0, n.d.

RASS-PAL = Richmond Agitation-Sedation Scale modified for Palliative Care inpatients.

PS = Palliative Sedation.

NSCLC = Non-small cell lung cancer.

ALS = Amyotrophic Lateral Sclerosis.

PPS = Palliative Performance Scale, version 2 [22].

HCP = Health Care Professional:

HCP1 = Palliative Care (PC) Physician.

HCP2 = Practice Support Nurse (PSN).

HCP3 = Bedside Nurse (BSN).

n.d. = assessment not done.

**Table 2:** Inter-rater correlations of RASS-PAL

	**“Initial” study modified RASS inter-rater ICC**	**RASS-PAL inter-rater ICC (including patient ID 10)**	**RASS-PAL inter-rater ICC (excluding patient ID 10)**
Time 1	0.90 (0.74, 0.96)	0.98 (0.93, 0.99)	0.98 (0.95, 1.00)
Time 2	0.95 (0.85, 0.98)	0.64 (0.29, 0.89)	0.84 (0.56, 0.95)
Time 3	0.76 (0.44, 0.92)	0.86 (0.60, 0.96)	0.94 (0.79, 0.98)
Time 4	0.95 (0.85, 0.98)	0.74 (0.38, 0.93)	0.97 (0.89, 0.99)
Time 5	0.89 (0.70, 0.97)	0.91 (0.67, 0.98)	0.98 (0.89, 1.00)

ICC = Intraclass correlation coefficient and 95% confidence intervals.

**Figure 2:**
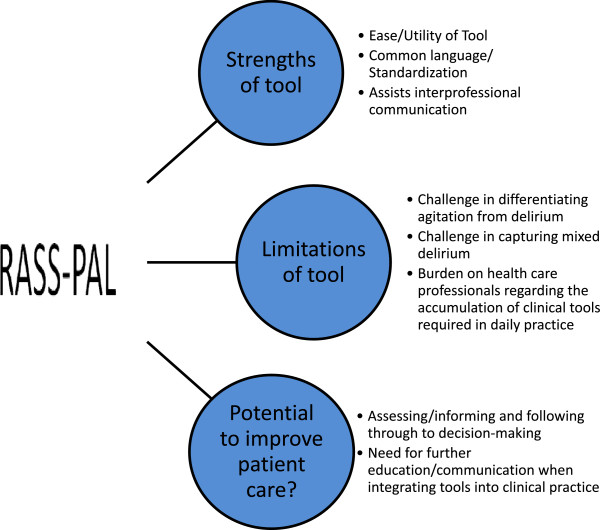
Model of RASS-PAL themes.

Thirteen interviews were conducted. Three primary themes (Figure 2) emerged from this data: (1) Strengths and (2) Limitations of the tool in the PCU; and (3) Potential to improve patient care. With respect to (1), ease or utility of the instrument was identified as was its ability to facilitate the use of a common language, definitions and understanding. Tables 3 and 4 provide qualitative quotes to support this analysis, as well as quotes for the other emerging themes. Professionals across all three disciplines expressed that the RASS-PAL was easy to use, simple and brief. Clearly articulated within many of these comments was the importance of ‘ease’ and simplicity in rolling out tools for HCPs to use on the PCU. While many clinical practice tools do not always define the concepts they are using, the RASS-PAL is considered a particularly strong tool, as each level of sedation or agitation is defined. Clinical advantages ascribed to the RASS-PAL generally related to how HCPs felt the tool could inform practice. Many identified the challenges of measuring and communicating clinical findings related to sedation and agitation levels in a standardized way in daily practice. Professionals felt that using the RASS-PAL tool created a common language for team members, which would also help standardize a working definition for sedation and agitation. This standardization was deemed very important because of the subjectivity in this area and the variability amongst team members of understandings and definitions of sedation and agitation on the PCU.

**Table 3:** Qualitative comments: strengths of utilising the RASS-PAL tool in palliative care inpatients

**Strengths of RASS-PAL tool**		
**Ease/Utility of tool**		
Palliative care physician	“…it was easy to score”	ID 1001
Nurse	“…the explanations were very good…they are quite easy to understand”	ID 1009
Nurse	“how you assess the patient, it’s….the degree of delirium or agitation is easier recognized when you’re following the protocol for this thing.	ID 1008
**Common language/Standardization**		
Palliative care physician	“that we’re all talking the same language right? …because what I consider something to be moderate or severe, someone else might not so it kind of delineates exactly what that is”	ID 1012
Nurse	“…if everyone follows this…then I think it would be easier if somebody says, “oh they are a -3 or +2” …you quickly know exactly where they are”	ID 1011
Palliative care physician	“I think it gives an objective number to what you’re observing, I think that’s its advantage, instead of just saying well you know, you just sort of generalize at times but this makes you kind of specify what you’re actually seeing”	ID 1002
**Assists interprofessional communication**		
Palliative care physician	“…it also gives you a language that different members of the care team could come to you and say, ‘This is what I want’, or ‘this is what I’m seeing’, ‘this is where we should go'”	ID 1010
Nurse	“…it’s also good because it would be easier also to transfer that information to the staff, to all the staff that will take care of this patient and also the doctors and the interdisciplinary team”	ID 1013
Palliative care physician	“…I think it’s potentially very useful in clinical care and for communication… among staff members…if a nurse is coming onto their shift and they look at this, they can see well…that they patient could have been quite agitated and they’ll be on their guard and need to be on the watch …”	ID 1002
Palliative care physician	“…better communication, better documentation, better understanding over time, so you can look back and…you can see how…things are progressing over time”	ID 1012

**Table 4:** Qualitative comments: limitations of utilising the RASS-PAL tool and its potential to improve patient care

**Limitations of RASS-PAL tool**		
**Challenge in differentiating agitation from delirium**		
Nurse	“…when you called their name I think some people (and they woke up), some people didn’t really know how to rate them. They might not have been agitated but they were very confused and….sometimes I had trouble doing that.”	ID 1001
Palliative care physician	“…but when you’re dealing with an agitated patient, there’s so…for example, hyperactive delirium, there’s so many other components then just motor agitation or something and that you can’t really assess you know and it change so fast you now…”	ID 1005
**Challenge in capturing mixed delirium**		
Palliative care physician	“I found it difficult to score because …in the time-frame that we were looking at them they had elements of both…so mixed delirium could be more difficult to score.”	ID 1007
Palliative care physician	“…but I don’t think it was as easy to do…when they’re calm or like seem to be calm and then you know that they were just really agitated a little while ago.”	ID 1002
**Burden on health care professional**		
Nurse	“If we were going to add these two forms of evaluation…that’s a lot of paperwork for me to do that with 4 different patients to have 3 different surveys throughout the day, that is very hard…very hard.”	ID 1004
**Potential to improve patient care?**		
**Assessing, informing, following-through**		
Nurse	“I think if staff follow through and medicate patients appropriately, then it’s good.”	ID 1001
Palliative care physician	“I think it informs us a lot better and guides decision making, in terms of whether somebody is going to get medication or not.”	ID 1007
Nurse	“it’s a good guide….it’ll improve in the sense that it’s a good guide, give us an idea, but not as far as hands-on care.. we’re still…not going to go to a paper.”	ID 1009
Nurse	“I would say it improves patient care for sure because you’re assessing the degree of delirium or agitation and….you’re paying attention.”	ID 1008
**Need for further education/communication when integrating tools into practice**		
Nurse	“…we’d need …a bit more education if it’s going to be used on a regular basis like our other paperwork.”	ID 1009
Palliative care physician	“…staff if they don’t understand how to use it, they’re not going to use it, so they need to be educated. And some staff will fill it out, but then you’ll look and they won’t give meds.”	ID 1002

With respect to (2), limitations in using it to monitor agitated or mixed-type delirium were identified. One physician cautioned that the motor ‘agitation’ as assessed by the RASS-PAL at a single point in time was not a direct measure for ‘agitated delirium’. A longer period of observation was needed to enable identification of other delirium diagnostic features. Professionals reported that it was much more challenging to use the RASS-PAL in patients with mixed delirium. Study field notes showed that nurses struggled to rate a patient with mixed delirium who was observed to be drowsy. As another limitation, some nursing professionals expressed concern that utilizing such an instrument would add to their burden of daily completion and concomitant paperwork required for other instruments on the PCU as part of a recent local quality initiative i.e. ESAS [23] and Nu-DESC [15].

The majority of HCPs felt that the tool had the potential to improve patient care. However, some stipulated that patient care would only improve if the tool was used to inform clinical therapies and practices within the team.

“Yeah I mean…in so much as it can create a common language….a tool in of itself cannot change patient care. People using the tool will if they use common language together so that they’re on the same page about the sedation targets and results and…use that language as a means of titrating their therapy”. **(Palliative Care Physician – ID 1010)**

All disciplines identified the need for a broader education initiative to better orientate them to the instrument.

## Discussion

This study provides preliminary validity evidence for the use of the RASS-PAL in assessing the level of sedation and agitation in palliative care inpatient populations, more specifically in the context of PS. Consistent with studies of the original RASS [5,6], we demonstrate that the RASS-PAL has good psychometric properties with high inter-rater reliability [24] when used by HCPs on a PCU. Similarly a modified RASS (with different modifications as compared with the RASS-PAL) used in Spanish patients with advanced cancer also demonstrated very good inter-rater reliability, especially when used by experienced professionals [13].

There were general improvements in reliability utilizing the RASS-PAL as compared with our initial modified version of the RASS. Evidence for face validity was noted, although we recognize that emerging understandings of the concept of validity place less emphasis on this property. The RASS instrument modifications at the beginning of the study and following the “initial” study, and qualitative and quantitative data showing general agreement amongst HCPs on the role of the RASS-PAL, provide evidence for construct validity.

Professionals said that they were often uncomfortable with the subjectivity and ambiguity that characterize discussions between team members about sedation and agitation. It is clear that they are interested in finding working definitions of levels of sedation and agitation, which can be used across interprofessional teams, to improve communication and patient care [25-27]. The RASS-PAL tool offers a framework that can be used across interprofessional teams to effectively communicate and collaborate together as they develop a plan of care. Ensuring strong and clear communication about sedation, agitation and delirium across the interprofessional team at all times, particularly at high-risk times in terms of shift changes and handovers in care, is absolutely critical in ensuring that patients receive the best possible care [28].

Our findings differ in some key respects from previous studies [9,13]. Chester et al. reported a modified RASS which could be utilized for daily delirium screening, but their tool incorporated an assessment of attention as part of the instrument modification [9]. Lack of attention is increasingly recognized as a key feature of delirium [29]. Our modifications did not include this feature, which may explain some of the difference in results between our study and theirs.

Agitation is not a delirium specific sign and underlying causes for agitation in the ICU setting may be different than in a PCU setting, such as ‘fighting the ventilator’ [27,30-33]. In addition, the assessment of patients with mixed delirium remains challenging as a single assessment and corresponding score is not adequate [6]. Sessler described this as a limitation to the original RASS where patients appeared to be sleeping or sedated but responded to auditory or physical stimulation violently [5].

In our study, concerns were expressed on using the RASS-PAL for monitoring patients with delirium because of the fluctuating nature of delirium as the instrument provides in essence a ‘snap-shot’ assessment. As refractory agitated delirium is common at the end of life [14], the RASS-PAL could however play a role in providing justification for using a rescue antipsychotic dose in agitated delirium and assessing its short-term impact. The RASS and RASS-PAL are very different to delirium tools such as the Nu-DESC [15], DRS-R-98 [34], and MDAS [35] which measure delirium over an extended time-frame. The Agitation Distress Scale, designed to measure agitation distress in delirious patients at the end of life, is also rated over a 12-hour time period [33]. Delirium evaluation in the ICU setting routinely comprises the CAM-ICU in addition to the RASS [36]. Therefore, the use of the RASS-PAL to monitor or screen for delirium in the palliative care setting warrants further research before it is more broadly recommended.

Professionals noted that the RASS-PAL had the potential to improve patient care. They highlighted the possibility for it to standardize practice, enhance communication and monitoring, and encourage follow through into decision-making. Future studies should examine the full application and implementation of how the RASS-PAL is used to inform clinical decision-making as this would offer a stronger understanding about how the RASS-PAL tool actually improves patient care. The issue of the instrument adding burden to nursing care warrants discussion. It appears the source of burden is not the RASS-PAL itself, but rather the overall use of instruments on the PCU. If tools such as the RASS-PAL are completed but not used by the clinical team to inform practices, professionals will regard these tools as one more piece of paper, required, but not useful. This echoes other studies which have shown that nurses may feel that scales are time consuming and have unclear relevance for their practice and patient care [37].

Contrary to initial studies where the original RASS was administered with minimal training [5,6] our study participants generally felt that significant formal education was required on how to integrate the RASS-PAL into clinical practice [4]. It is unlikely that the minor modifications of the RASS-PAL from the original RASS contributed to this. One possible explanation could relate to ICU nurses being more accustomed to such assessments where it is common practice to stimulate patients in order to assess sedation level in routine daily practice [26], but it is noteworthy that an educational strategy was later utilized for the large-scale implementation of the RASS [38]. Education for RASS-PAL implementation needs to emphasize both logistical and procedural elements of using the tool, as well as broader understandings of how varying HCPs perceive the tool informing their practice as well as other team members. Education strategies should be integrated education programs rather than one-time event [28,39]. This would promote a broader understanding of how the RASS-PAL would inform practice on multiple levels.

This study highlights the merits of mixed methods research approaches. As Farquhar et al. recently noted it is an approach that is “particularly valuable in palliative care research, where the majority of interventions are complex” [40]. Drawing on a mixed method design offered an opportunity to examine the process of evaluating and identifying sedation and agitation, and the experience of HCPs in applying the tool in practice.

There are several limitations to this study. This was a small pilot study which may limit the generalizability of the results. Notwithstanding this, it does raise some important issues and contributes to building validity evidence for the RASS-PAL. Not all aspects of validity evidence required to fully examine construct validity were explored [7,41]. On multiple occasions it was not possible for all three raters to be present for each time point, reflecting the real-life challenges of research on a PCU. As with other sedation instruments [5,42], this study did not test the responsiveness of the RASS-PAL instrument in detecting important changes in sedation over time, nor did it specifically evaluate how utilizing the RASS-PAL will change PS practice.

## Conclusions

We found that the RASS-PAL has high inter-rater reliability and appears useful in monitoring sedation level in patients receiving PS. It appears to have potential to standardize PS practice and improve interprofessional patient care. Future studies should validate the RASS-PAL against other sedation monitoring instruments and assess for other sources of validity evidence in this patient population and context of care. Although it does not specifically screen for delirium, the RASS-PAL provides a snapshot assessment in time for agitation. Further research on the feasibility of using the RASS-PAL to monitor established delirium in palliative care patients is required. Further research is also needed on its large-scale implementation in palliative care settings with multifaceted educational strategies and evaluation of ongoing reliability.

## Competing interests

The authors declare that they have no competing interests.

## Authors’ contributions

SB participated in the design of the study, performed the qualitative statistical analysis and drafted the manuscript. PG participated in the qualitative statistical analysis, the interpretation of this data and helped to draft the manuscript. MY conducted participant interviews, participated in the study coordination and performed the qualitative statistical analysis. TZ performed the quantitative statistical analysis. SZ conducted participant interviews and participated in the study coordination. JP conceived of the study, and participated in its design and helped to draft the manuscript. All authors read and approved the final manuscript.

## Pre-publication history

The pre-publication history for this paper can be accessed here:

http://www.biomedcentral.com/1472-684X/13/17/prepub

## Supplementary Material

Additional file 1: Figure S1 Richmond Agitation-Sedation Scale - Palliative version (RASS-PAL). Click here for file

Additional file 2: Table S2 Procedure for RASS-PAL Assessment. Click here for file
