# Supplementary material for: The Richmond Agitation-Sedation Scale modified for palliative care inpatients (RASS-PAL): a pilot study exploring validity and feasibility in clinical practice
Source: BMC Palliat Care. 2014 Mar 31;13:17. doi: 10.1186/1472-684X-13-17 (PMC3997822; doi:10.1186/1472-684X-13-17)
Supplement: Additional file 1: Figure S1 — Richmond Agitation-Sedation Scale - Palliative version (RASS-PAL). [file 1472-684X-13-17-S1.doc]

### Additional file 1: Figure S1- Richmond Agitation-Sedation Scale - Palliative version (RASS-PAL)

| **Score** | **Term** | **Description** |
| --- | --- | --- |
| +4 | Combative | Overtly combative, violent, immediate danger to staff (e.g. throwing items); +/- attempting to get out of bed or chair |
| +3 | Very agitated | Pulls or removes lines (e.g. IV/SQ/Oxygen tubing) or catheter(s); aggressive, +/- attempting to get out of bed or chair |
| +2 | Agitated | Frequent non-purposeful movement, +/- attempting to get out of bed or chair |
| +1 | Restless | Occasional non-purposeful movement, but movements not aggressive or vigorous |
| 0 | Alert and calm |  |
| -1 | Drowsy | Not fully alert, but has sustained awakening (eye-opening/eye contact) to *voice* **(10 seconds or longer)**  Verbal Stimulation |
| -2 | Light sedation | Briefly awakens with eye contact to *voice* **(less than** **10 seconds)** |
| -3 | Moderate sedation | Any movement (eye or body) or eye opening to *voice* **(but no eye contact)** |
| -4 | Deep sedation | No response to voice , but any movement (eye or body) or eye opening to *stimulation by light touch*  Gentle Physical Stimulation |
| -5 | Not rousable | No response to *voice or stimulation by light touch* |

### 
