# Supplementary material for: The Richmond Agitation-Sedation Scale modified for palliative care inpatients (RASS-PAL): a pilot study exploring validity and feasibility in clinical practice
Source: BMC Palliat Care. 2014 Mar 31;13:17. doi: 10.1186/1472-684X-13-17 (PMC3997822; doi:10.1186/1472-684X-13-17)
Supplement: Additional file 2: Table S2 — Procedure for RASS-PAL Assessment. [file 1472-684X-13-17-S2.docx]

### Additional file 2: TableS2- Procedure for RASS-PAL Assessment

| 1. Observe patient for **20 seconds.** |  |
| --- | --- |
| a. Patient is alert, restless, or agitated **for more than 10 seconds**  **NOTE:** If patient is alert, restless, or agitated for less than 10 seconds and is otherwise drowsy, then score patient according to your assessment for the majority of the observation period | Score 0 to +4 |
| 2. If not alert, greet patient and call patient by name and *say* to open eyes and look at speaker. |  |
| b. Patient awakens with sustained eye opening and eye contact **(10 seconds or longer).** | Score -1 |
| c. Patient awakens with eye opening and eye contact, but not sustained **(less than** **10 seconds).** | Score -2 |
| d. Patient has any eye or body movement in response to voice but no eye contact. | Score -3 |
| 3. When no response to verbal stimulation, physically stimulate patient by light touch e.g. gently shake shoulder. |  |
| e. Patient has any eye or body movement to gentle physical stimulation. | Score -4 |
| f. Patient has no response to any stimulation. | Score -5 |
|  |  |
